# Persistent Hypochloremia Is Associated with Adverse Prognosis in Patients Repeatedly Hospitalized for Heart Failure

**DOI:** 10.3390/jcm12041257

**Published:** 2023-02-05

**Authors:** Yuji Nozaki, Akiomi Yoshihisa, Yu Sato, Himika Ohara, Yukiko Sugawara, Satoshi Abe, Tomofumi Misaka, Takamasa Sato, Masayoshi Oikawa, Atsushi Kobayashi, Takayoshi Yamaki, Kazuhiko Nakazato, Yasuchika Takeishi

**Affiliations:** 1 Department of Cardiovascular Medicine, Fukushima Medical University, Fukushima 960-1247, Japan; 2 Department of Clinical Laboratory Sciences, Fukushima Medical University, Fukushima 960-1295, Japan

**Keywords:** hypochloremia, serum chloride, heart failure, cardiac death, all-cause death

## Abstract

Background: Hypochloremia reflects neuro-hormonal activation in patients with heart failure (HF). However, the prognostic impact of persistent hypochloremia in those patients remains unclear. Methods: We collected the data of patients who were hospitalized for HF at least twice between 2010 and 2021 (n = 348). Dialysis patients (n = 26) were excluded. The patients were divided into four groups based on the absence/presence of hypochloremia (<98 mmol/L) at discharge from their first and second hospitalizations: Group A (patients without hypochloremia at their first and second hospitalizations, n = 243); Group B (those with hypochloremia at their first hospitalization and without hypochloremia at their second hospitalization, n = 29); Group C (those without hypochloremia at their first hospitalization and with hypochloremia at their second hospitalization, n = 34); and Group D (those with hypochloremia at their first and second hospitalizations, n = 16). Results: a Kaplan–Meier analysis revealed that all-cause mortality and cardiac mortality were the highest in Group D compared to the other groups. A multivariable Cox proportional hazard analysis revealed that persistent hypochloremia was independently associated with both all-cause death (hazard ratio 3.490, *p* < 0.001) and cardiac death (hazard ratio 3.919, *p* < 0.001). Conclusions: In patients with HF, prolonged hypochloremia over two hospitalizations is associated with an adverse prognosis.

## 1. Introduction

Chloride is an important electrolyte involved in blood pressure maintenance, neurohormonal activation, nociception, intracellular electrolyte transport, and cell volume regulation, and it is also important in cardiac and renal signaling [1]. Chloride is also a regulator of electrolyte and fluid reabsorption in the kidney and is involved in the regulation of renin secretion, tubuloglomerular feedback, and diuretic sensitive sodium channels [2,3]. Diuretics are used to treat dyspnea or edema in heart failure (HF) patients, but the latest clinical guidelines have reported that diuretics do not reduce mortality [4,5,6,7]. Diuretic use can cause hypochloremia [8], and furthermore, hypochloremia itself has recently been reported to be associated with mortality because it activates the renin-angiotensin-aldosterone system and causes loop diuretic tolerance [9,10,11,12]. The association of hypochloremia and the usage of loop diuretics with the risk of death in patients with HF has been reported: electrolyte imbalance after treatment with loop diuretics occurs in HF patients who require high-dose loop diuretics and results in increased mortality [13]. Serum chloride is a potential new therapeutic target in the pathophysiology of HF [2,14,15]. However, blood levels of chloride do not remain constant over the course of treatment [11,16]. Persistent or newly developed hypochloremia two weeks after the initiation of HF treatment is associated with reduced survival [16]. However, the impact of hypochloremia lasting for a longer duration is not clear. Because of a growing and aging population, the total number of heart failure patients still continues to rise [17,18,19]. In addition, each re-hospitalization sometimes requires intensive care, which is problematic due to the enormous medical costs involved [20]. The present study aimed to investigate the significance of persistent hypochloremia in patients who had been hospitalized twice due to HF by determining the association between changes in the blood levels of chloride and the risk of mortality.

## 2. Materials and Methods

### 2.1. Subjects and Protocol

**Figure 1:**
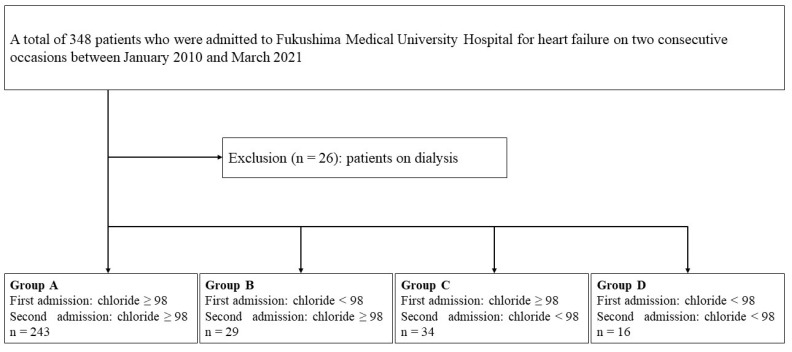
Patient flowchart.

This study was an observational study. A patient flowchart is shown in Figure 1. A total of 348 patients who were admitted to Fukushima Medical University Hospital for HF on two occasions between January 2010 and March 2021 were enrolled. The diagnosis of HF was made by the attending cardiologists based on the HF guidelines [7,21,22,23,24]. Among them, a total of 26 patients on dialysis were excluded. The remaining 322 patients were included in the analysis. Hypochloremia was defined as levels of serum chloride of <98 mmol/L, in accordance with a previous study focusing on hypochloremia in patients with HF [13]. The patients were divided into four groups based on the changes in the presence/absence of hypochloremia: Group A (n = 243, 75.4%), those without hypochloremia at the first and the second hospitalizations; Group B (n = 29, 9.0%), those with hypochloremia at the first hospitalization and without hypochloremia at the second hospitalization; Group C (n = 34, 10.6%), those without hypochloremia at the first hospitalization and with hypochloremia at the second hospitalization; and Group D (n = 16, 5.0%), those with hypochloremia at the first and the second hospitalizations. Groups B and C were defined as transient hypochloremia. Group D was defined as persistent hypochloremia. The patient characteristics and prognoses after the second discharge were compared among the four groups. The patient characteristics included demographic data at discharge after the first hospitalization and the laboratory data at the first and second hospitalizations. The laboratory data were collected at hospital discharge. The type of HF was classified as HF with reduced, mildly reduced, and preserved ejection fraction (those with left ventricular ejection fraction of <40%, 40–49%, and ≥50%, respectively). Ischemic HF was defined as HF caused mainly by coronary artery disease as adjudicated by the attending physicians. The primary endpoints were all-cause death and cardiac death after the second discharge. Cardiac death was defined as death due to worsening HF, acute coronary syndrome, or ventricular fibrillation [25,26,27].

The present study complies with the Declaration of Helsinki and the STROBE (Strengthening the Reporting of Observational studies in Epidemiology) statement [28,29]. The study protocol was approved by the Ethics Committee of Fukushima Medical University, and all the patients enrolled in the study provided written informed consent.

### 2.2. Methods for Measuring Serum Chloride

The measurement device used was Atellica Solution CH930 (Siemens Healthineers, Tokyo, Japan). The measurement principle used was the electrode measurement method, which uses indirect integrated multisensory technology to measure electrolytes in specimens. Indirect integrated multisensory technology has an ion-selective electrode (sodium, potassium, and chloride) and a reference electrode. The potential difference between these electrodes is measured, and the concentration of each ion is calculated using Nernst’s formula [30].

### 2.3. Statistical Analysis

The continuous variables, except for the levels of hemoglobin at the first and second hospitalizations, were considered to be non-normally distributed by the Shapiro–Wilk test and were presented as medians [25th and 75th percentile]. The normally distributed continuous variables were expressed as means ± standard deviation. The categorical variables were expressed as numerical values (percent). The non-normally and normally distributed variables were compared using the Kruskal–Wallis test followed by the Steel–Dwass test and the one-way analysis of variance followed by the Tukey test, respectively. The chi-square test was used for the comparisons of the categorical variables. The cumulative incidence of the primary endpoints was estimated via Kaplan–Meier analysis, and the differences were evaluated by the log-rank test. The effect of the change in hypochloremia as a predictor of the primary endpoints was evaluated by Cox proportional hazards analysis. In all the analyses, *p* values of < 0.05 were considered statistically significant. The Kruskal–Wallis and Steel–Dwass tests were performed using EZR version 1.55, the graphical user interface of R (The R Foundation for Statistical Computing, Vienna, Austria) [31]. All the other analyses were performed using IBM SPSS Statistics version 28 (IBM, Armonk, NY, USA).

## 3. Results

Comparisons of the patient characteristics are shown in Table 1. Group D showed the significantly oldest age (Group A, B, C, and D; 72, 64, 75, and 77 years, Kruskal–Wallis *p* = 0.027, *p* < 0.05 vs. Group B) and the highest prevalence of atrial fibrillation (44%, 48.3%, 52.9%, and 87.5%, *p* = 0.008). There were no statistical differences in the use of medications, including loop diuretics, among the four groups. There were no differences in the left ventricular ejection fraction and the prevalence of the type of HF among the four groups. In the laboratory data, Group D showed the significantly lowest levels of hemoglobin at the second hospitalization (11.7, 11.2, 11.5, and 10.1 g/dL, Kruskal–Wallis *p* = 0.025, *p* < 0.05 vs. Group A). The levels of sodium and chloride at the first and second hospitalizations showed significant intergroup differences (*p* < 0.001). 

**Figure 2:**
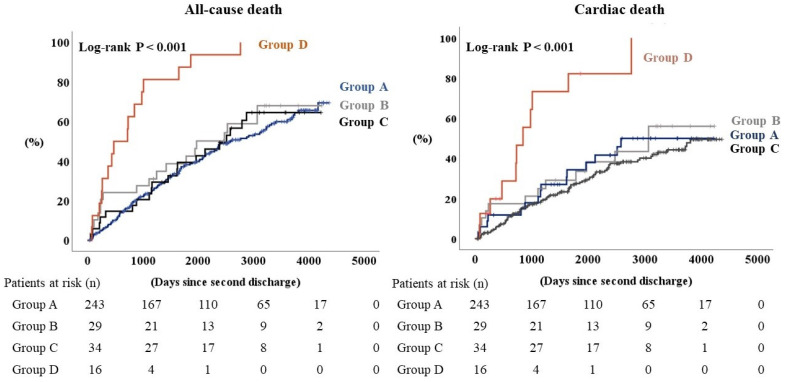
Kaplan–Meier analysis for all-cause death and cardiac death when heart failure patients are sorted into four groups according to blood levels of chloride.

During the follow-up period of a median 1694 days, a total of 171 all-cause deaths, including 119 cardiac deaths, were identified. All of the other patients were censored. The Kaplan–Meier analysis showed that both the all-cause and the cardiac deaths were highest in Group D, in which all the patients died during the follow-up period (Figure 2, log-rank *p* < 0.001, respectively). In the Cox proportional hazards analysis (Table 2), Group D had a hazard ratio of 3.490 for all-cause death and 3.919 for cardiac death after adjustment for age, sex, body mass index, atrial fibrillation, and serum sodium level at the second HF hospitalization, compared with Group A as a reference. There was no significant difference in Group B or Group C, compared to Group A.

## 4. Discussion

To the best of our knowledge, this is the first study to demonstrate that persistent hypochloremia in two HF hospitalizations is a high risk factor for cardiac and all-cause death. Although there have been reports of an increased risk of death in patients with hypochloremia during a single HF hospitalization [2,3,12,16,32,33], we observed changes in the serum levels of chloride between two HF hospitalizations in the present study. The transient hypochloremia group did not have an increased risk of mortality. On the other hand, all the patients with persistent hypochloremia between the two hospitalizations died during the later follow-up period.

The renal macula densa is sensitive to chloride and promotes renin secretion when it senses a decrease in the urinary chloride concentration [34]. Thus, hypochloremia activates the renin-angiotensin-aldosterone system [11,34]. The prevalence of atrial fibrillation was significantly higher in Group D in this study. This may be due to the activation of the renin-angiotensin-aldosterone system by persistent hypochloremia, causing atrial remodeling, which may have influenced the occurrence of atrial fibrillation [35]. Hypochloremia decreases the activity of chloride channels in the heart and can cause arrhythmic attacks as well as myocardial ischemia [36]. In addition, genetic studies have shown that amino acid substitutions in the chloride potential-gated channel Ka gene affect the renal chloride ion channels and are associated with increased HF independently of myocardial damage [14,37].

Recent studies have identified a specific family of serine-threonine kinases called With-No-Lysine kinases (WNKs) [38]. WNKs regulate the transporters through which loop diuretics and thiazide diuretics act [16,38]. Primarily, WNK3 regulates the activation of sodium-potassium-chloride co-transporters, the target of loop diuretics, and WNK4 regulates the activation of sodium-chloride co-transporter receptors, the target of thiazide diuretics [38,39]. Serum chloride binds directly to WNKs and inhibits their phosphorylation, thereby reducing the efficiency of the transporter reabsorption [16,39]. However, when WNKs sense hypochloremia, they are reported to cause upregulation of the sodium-potassium-chloride co-transporter and the sodium-chloride co-transporter receptor to increase the efficiency of chloride reabsorption [2,11,15,39]. Loop diuretics and thiazide diuretics exert their diuretic effect by inhibiting the sodium-potassium-chloride and sodium-chloride co-transporters. Therefore, hypochloremia is a cause of diuretic tolerance [2,11,14,15]. In this study, approximately 90% of the patients were receiving loop diuretics. This high use of loop diuretics was consistent with a national registry of HF [40]. As to the dose of loop diuretics, a prior study reported that there is a negative relationship between the levels of serum chloride and the dose of loop diuretics [10]. Although it is a matter of speculation, these results suggested that the dose of loop diuretics may have differed among the four groups of our study. From the results of our study, the association between the dose of loop diuretics and persistent hypochloremia over two hospitalizations should be elucidated in further research. 

The prolonged duration of hypochloremia with chronic high-dose loop diuretics may act on WNKs and cause the upregulation of sodium-potassium-chloride co-transporters and sodium-chloride co-transporter receptors [16,38]. This may lead to tolerance to loop diuretics, and the use of even higher doses of loop diuretics may cause a malignant cycle of persistent hypochloremia. There was no association with the risk of mortality in patients with transient hypochloremia, suggesting that hypochloremia may cause transporter upregulation and tolerance to loop diuretics in the period leading up to a second HF hospitalization and may be associated with the risk of mortality [6,13,16,41]. This suggests that tolerance to loop diuretics due to transporter upregulation is caused by long-lasting hypochloremia [12].

Therefore, it is suggested that intervention for hypochloremia at the time of initial HF hospitalization may have clinical significance [13]. Reportedly, loop diuretics increase chloride excretion 20-fold [10]. Acetazolamide alone has poor diuretic and sodium excretion capacity, but in combination with a loop diuretic, acetazolamide may be able to increase the diuretic effect efficiently [42,43,44]. In addition, a 5% reduction in the loss of chloride by acetazolamide has been reported [45]. The addition of other diuretics, such as vasopressin receptor antagonists, which do not cause renin-angiotensin-aldosterone system activation, may reduce the use of loop diuretics [10]. Further investigation into the pathophysiology of hypochloremia and its treatment for HF is warranted. 

## 5. Limitations

First, this research was performed in a single center with a relatively small number of patients and included a single ethnicity. The mortality risk associated with hypochloremia should be validated in a larger HF population and in populations of different ethnicities. Second, it was not an aim of the present study to clarify how to treat HF patients with hypochloremia. Third, we did not examine the dose of loop diuretics and changes in medications in this study. Fourth, there were few data on novel medications for HF, such as angiotensin receptor neprilysin inhibitors, ivabradine, and sodium–glucose co-transporter 2 inhibitors in the study period.

## 6. Conclusions

In patients with HF, prolonged hypochloremia over two hospitalizations is associated with an adverse prognosis.

## Figures and Tables

**Table 1:** Patient characteristics.

	Total (n = 322)	Group A (n = 243)	Group B (n = 29)	Group C (n = 34)	Group D (n = 16)	*p* Value
Demographic data						
Age, years	72 [61, 80]	72 [60, 81]	64 [53.5, 75]	75 [61.5, 81]	76.5 [69.5, 80] †	0.027
Male sex, n (%)	203 (63)	154 (63.4)	19 (65.5)	20 (58.8)	10 (62.5)	0.950
BMI, kg/m^2^	22.2 [19.8, 24.6]	22.3 [20.2, 24.8]	22.5 [19.1, 25.2]	20.8 [17.4, 24] *	20.7 [17.6, 23.6]	0.046
SBP, mmHg	120 [102, 143]	121 [104, 142]	120 [93, 150]	120 [102, 164]	112 [91, 133]	0.486
Hypertension, n (%)	253 (78.6)	191 (78.6)	24 (82.8)	26 (76.5)	12 (76.5)	0.916
Diabetes mellitus, n (%)	162 (50.3)	119 (49)	18 (62.1)	14 (41.2)	11 (68.8)	0.165
Dyslipidemia, n (%)	255 (79.2)	186 (76.5)	25 (86.2)	29 (85.3)	15 (93.8)	0.193
Atrial fibrillation, n (%)	153 (47.5)	107 (44)	14 (48.3)	18 (52.9)	14 (87.5)	0.008
CAD, n (%)	106 (32.9)	82 (33.7)	10 (34.5)	10 (29.4)	4 (25)	0.861
RAS inhibitors, n (%)	253 (78.6)	193 (79.4)	21 (72.4)	27 (79.4)	12 (75)	0.827
Beta blockers, n (%)	264 (82)	199 (81.9)	24 (82.8)	28 (82.4)	13 (81.3)	0.999
Loop diuretics, n (%)	285 (88.5)	210 (86.4)	26 (89.7)	33 (97.1)	16 (100)	0.133
LVEF, %	46.3 [34, 60.5]	45.8 [33.7, 60]	44.4 [32, 58.7]	49.2 [34, 62]	55.4 [36.6, 63]	0.563
Type of HF, n (%) HFrEF HFmrEF HFpEF	116 (36) 62 (19.3) 144 (44.7)	86 (35.4) 50 (20.6) 107 (44)	13 (44.8) 6 (20.7) 10 (34.5)	11 (32.4) 6 (17.6) 17 (50)	6 (37.5) 0 (0) 10 (62.5)	0.400
Ischemic HF, n (%)	78 (24.2)	61 (25.1)	7 (24.1)	8 (23.5)	2 (12.5)	0.727
NYHA class 3 or 4, n (%)	20 (6.2)	15 (6.2)	2 (6.9)	1 (2.9)	2 (12.5)	0.629
Time between 1st and 2nd hospitalizations, days	295 [93, 948]	279 [93, 895]	267 [78, 866]	728 [222, 1129]	236 [70.5, 621]	0.081
Laboratory data						
BNP, pg/mL at 1st hospitalization	412 [189, 803]	395 [178, 779]	390 [147, 969]	435 [194, 707]	574 [261, 1475]	0.600
BNP, pg/mL at 2nd hospitalization	480 [210, 930]	484 [223, 882]	514 [146, 997]	425 [179, 1084]	488 [209, 1500]	0.815
Hemoglobin, g/dL at 1st hospitalization	12.5 ± 2.2	12.7 ± 2.2	12 ± 2.2	11.9 ± 1.7	11.2 ± 2	0.017
Hemoglobin, g/dL at 2nd hospitalization	11.6 ± 2.3	11.7 ± 2.3	11.2 ± 2.1	11.5 ± 1.5	10.1 ± 2 *	0.025
eGFR, mL/min/1.73 m^2^ at 1st hospitalization	53.1 [39, 70.9]	53.1 [41.2, 70.2]	48.5 [33.3, 67.9]	60.1 [37.3, 77.1]	48.4 [34.1, 67.3]	0.513
eGFR, mL/min/1.73 m^2^ at 2nd hospitalization	46.5 [33.8, 61.5]	46.5 [34, 61]	52 [37.5, 71]	42.8 [30.3, 67.1]	42.7 [26, 55.7]	0.383
Sodium, mmol/L at 1st hospitalization	139 [137, 141]	140 [138, 141] †‡	135.5 [130.5, 137] *‡	138 [136, 142] *†	134 [131, 138] *†‡	<0.001
Sodium, mmol/L at 2nd hospitalization	139 [137, 141.5]	140 [138, 142] †‡	140 [136, 141] *‡	134 [129.5, 137.5] *†	134.5 [132, 140] *†‡	<0.001
Potassium, mmol/L at 1st hospitalization	4.2 [3.9, 4.5]	4.1 [3.9, 4.5]	4.3 [3.8, 5.2]	4.3 [4.1, 4.6]	4.2 [4.1, 4.9]	0.235
Potassium, mmol/L at 2nd hospitalization	4.1 [3.8, 4.5]	4.1 [3.8, 4.5]	4.3 [3.8, 4.6]	4.1 [3.8, 4.6]	4.1 [3.7, 4.3]	0.795
Chloride, mmol/L at 1st hospitalization	103 [100, 106]	104 [102, 107] †‡	96 [93, 97] *‡	103 [100, 106] *†	94.5 [90.5, 96] *†‡	<0.001
Chloride, mmol/L at 2nd hospitalization	102 [99, 106]	104 [101, 107] †‡	103 [100, 104] *‡	95 [93, 96] *†	93.5 [90.5, 95.5] *†‡	<0.001

Group A (n = 243, 75.4%), those without hypochloremia at the first and the second hospitalizations; Group B (n = 29, 9.0%), those with hypochloremia at the first hospitalization and without hypochloremia at the second hospitalization; Group C (n = 34, 10.6%), those without hypochloremia at the first hospitalization and with hypochloremia at the second hospitalization; and Group D (n = 16, 5.0%), those with hypochloremia at the first and the second hospitalizations. BMI, body mass index; BNP, B-type natriuretic peptide; CAD, coronary artery disease; eGFR, estimated glomerular filtration rate; HF, heart failure; HFmrEF, HF with mildly reduced ejection fraction; HFpEF, HF with preserved ejection fraction; HFrEF, HF with reduced ejection fraction; LVEF, left ventricular ejection fraction; NYNA, New York Heart Association; RAS, renin-angiotensin system; SBP, systolic blood pressure. Non-normally and normally distributed variables are expressed as medians [25th and 75th percentile] and means ± standard deviation, respectively. Non-normally and normally distributed variables are compared using the Kruskal–Wallis test followed by the Steel–Dwass test and the one-way analysis of variance followed by the Tukey test, respectively. The chi-square test was used for the comparisons of categorical variables. * *p* < 0.05 vs. group A, † *p* < 0.05 vs. group B, and ‡ *p* < 0.05 vs. Group C.

**Table 2:** Cox proportional hazard analysis after 2nd hospitalization.

	HR (95% CI)	*p* Value
All-cause death (event n = 171/322 patients)		
Group D (vs. Group A) unadjusted	4.925 (2.892–8.387)	<0.001
Group D (vs. Group A) Model 1	3.866 (2.248–6.649)	<0.001
Group D (vs. Group A) Model 2	3.490 (1.957–6.224)	<0.001
Group C (vs. Group A) unadjusted	1.114 (0.695–1.788)	0.654
Group B (vs. Group A) unadjusted	1.243 (0.758–2.038)	0.390
Cardiac death (event n = 119/322 patients)		
Group D (vs. Group A) unadjusted	4.841 (2.548–9.198)	<0.001
Group D (vs. Group A) Model 1	3.846 (1.877–7.879)	<0.001
Group D (vs. Group A) Model 2	3.919 (1.804–8.512)	<0.001
Group C (vs. Group A) unadjusted	1.252 (0.722–2.172)	0.424
Group B (vs. Group A) unadjusted	1.348 (0.751–2.420)	0.318

Model 1: adjusted for age and sex. Model 2: adjusted for atrial fibrillation, age, body mass index, hemoglobin of second hospitalization, sex, sodium of second hospitalization. CI, confidence interval; HR, hazard ratio.

## Data Availability

The data presented in this study are available on request from the corresponding author.
